# Impact of cerebral oxygenation-guided resuscitation during immediate postnatal transition on brain injury and brain growth detected by MRI in very preterm neonates: a secondary outcome analysis of the multicenter randomized phase 3 clinical COSGOD III trial

**DOI:** 10.1186/s13052-026-02216-7

**Published:** 2026-02-24

**Authors:** Marlene Hammerl, Christina Schreiner, Elke Griesmaier, Ursula Kiechl-Kohlendorfer, Daniel Pfurtscheller, Alexander Avian, Sebastian Tschauner, Katharina Goeral, Julia Buchmayer, Gianluca Lista, Ilaria Stucchi, Jenny Bua, Vera Neubauer, Gerhard Pichler

**Affiliations:** 1 Department of Pediatrics II (Neonatology), Medical University of Innsbruck, Anichstraße 35, Innsbruck, A-6020 Austria; 2 Division of Neonatology, Department of Pediatrics and Adolescent Medicine, Medical University of Graz, Graz, Austria; 3 Research Unit for Cerebral Development and Oximetry & Research Unit for Neonatal Micro - and Macrocirculation, Division of Neonatology, Department of Pediatrics and Adolescent Medicine, Medical University of Graz, Graz, Austria; 4 Institute for Medical Informatics, Statistics and Documentation, Medical University of Graz, Graz, Austria; 5 Department of Radiology, Division of Paediatric Radiology, Medical University of Graz, Graz, Austria; 6 Comprehensive Center for Pediatrics, Department of Pediatrics and Adolescent Medicine, Division of Neonatology, Intensive Care and Neuropediatrics, Full Member of ERN EpiCare, Medical University of Vienna, Vienna, Austria; 7 Division of Neonatology and Neonatal Intensive Care Unit, Ospedale dei Bambini Vittore Buzzi, Milano, Milan, Italy; 8 Neonatal Intensive Care Unit, Institute for Maternal and Child Health, IRCCS “Burlo Garofolo”, Trieste, Italy

## Abstract

**Background:**

The prospective, randomized-controlled multicenter COSGOD III trial was designed to investigate whether interventions guided by cerebral oxygen saturation (crSO_2_) measured with near-infrared spectroscopy (NIRS group) during the immediate postnatal transition increase survival without cerebral injury of very preterm infants compared with standard care alone (control group). The aim of this secondary outcome study was to compare brain injury and brain growth between the two groups, as assessed by magnetic resonance imaging (MRI) at near-term age.

**Methods:**

Patients from five centers participating in the COSGOD III trial, which performed near-term MRI, were evaluated for the presence of brain injury (intraventricular hemorrhage, cerebellar hemorrhage, white matter injury) and brain growth (transcerebellar/biparietal diameter, interhemispheric distance).

**Results:**

This study included 172 infants (86 per group) with a median gestational age of 28.4 weeks and a median birth weight of 1045 grams. The incidence of brain injury did not differ between the groups. Infants in the NIRS group exhibited a significantly larger median biparietal diameter (median [IQR] 78.6 [76.3–82.1] mm) compared to the control group (76.7 [74.7–80.0] mm). A trend toward a lower rate of abnormal biparietal diameter was observed in the NIRS group.

**Conclusions:**

Monitoring of crSO_2_ with dedicated treatment guidelines during the immediate postnatal transition had no effect on the incidence of brain injury assessed by MRI. The significantly larger biparietal diameter in the NIRS group may be indicative of enhanced brain development. The relationship between this structural advance in the NIRS group and long-term functional outcomes requires further investigation.

**Supplementary information:**

The online version contains supplementary material available at 10.1186/s13052-026-02216-7.

## Background

Preterm birth poses significant challenges, placing infants at increased risk of both short- and long-term morbidity [1–3]. Neonatologists strive to identify the factors that contribute to these risks and to implement strategies to mitigate them. A key area of focus is the immediate fetal-to-neonatal transition, a period characterized by profound physiological changes, including dramatic shifts in blood flow, blood pressure, and oxygenation [4]. These changes have a particularly pronounced impact on the brain, where the delicate balance between cerebral blood flow and oxygen delivery plays a vital role in ensuring neural integrity and cerebral development [5].

Consequently, the brain is particularly vulnerable to injury during this critical early period of life. Despite the complexity of this transition, standard neonatal resuscitation focuses only on monitoring heart rate (HR) and arterial oxygen saturation (SpO_2_) [6, 7].

The COSGOD III trial was a multinational, multicenter, randomized-controlled study to test the hypothesis that integrating near-infrared spectroscopy (NIRS) during the immediate fetal-to-neonatal transition for measuring cerebral regional tissue oxygen saturation (crSO_2_) and guiding intervention (respiratory support, supplemental oxygen administration and volume administration) in real-time improves outcomes in very preterm infants [8]. The intervention resulted in a non-significant improvement in survival without brain injury [8]. Notably, this primary study used cranial ultrasound, the widely established clinical routine, to assess brain injury. However, this tool has limited sensitivity to detect the full spectrum of preterm brain injury. Therefore, the true extent of potential differences between the study groups may have gone undetected. Magnetic resonance imaging (MRI) has been shown to have significant advantages over cranial ultrasound, particularly in detecting subtle white matter alterations and cerebellar hemorrhages. In addition, MRI provides insights into postnatal brain growth and cerebral development [9–11].

This secondary outcome study provides an extension of the COSGOD III findings. The focus of this study was a subgroup of infants who had undergone cerebral MRI examinations. The aim was to assess whether crSO_2_-guided interventions during the immediate postnatal transition of very preterm infants have an effect on brain injury and brain growth as determined by cerebral MRI at near-term age.

## Methods

### Study design

This study is a secondary outcome analysis of the COSGOD III trial conducted between October 2017 and February 2022. The COSGOD III trial investigated whether very preterm infants could benefit in terms of survival without cerebral injury (assessed by cerebral ultrasound) by adjusting interventions based on the crSO_2_-monitoring during immediate transition after birth (NIRS group). This approach was compared to standard care alone (control group).

Written parental consent was obtained before birth for inclusion into the COSGOD III trial. Randomization was conducted via a web-based randomization service (‘Randomizer for Clinical Trials’, https://www.randomizer.at/random/, Medical University of Graz, Austria). Intervention ended after the first 15 minutes of life. The study was approved by all local ethics committees. The COSGOD III trial was registered at Clinical Trials (NCT03166722). Cerebral injury assessed by MRI, if performed routinely, was defined as a secondary outcome parameter.

### COSGOD III protocol

After birth, routine monitoring with SpO2 and, optionally, ECG was applied to all infants. Within three minutes after birth and until 15 minutes of life, a crSO2 sensor was positioned on the left forehead.

In the control group, all delivery room interventions were performed according to local hospital guidelines and published neonatal resuscitation guidelines. In the NIRS group, interventions were guided by a stepwise approach: first, SpO2 was maintained within the target range (10th–90th centile) according to local and international guidelines. Only when SpO2 was within the target range were crSO2 values used to adjust therapy: if crSO2 fell below the lower reference limit, FiO2 was increased or respiratory support escalated; if crSO2 exceeded the upper reference limit, FiO2 or support was reduced. Values within the target range for more than one minute prompted a reduction in FiO2 or support. Detailed information on the protocol and results of the COSGOD III trial already have been published [8, 12].

### Study participants

Eleven centers participated in the COSGOD III trial. Out of these, five centers performed MRI analyses on a routine basis. Centers which only performed MRI scans for clinical purposes (e.g. determine the extent of a brain injury, birth asphyxia, neurological abnormalities) were excluded a-priori to avoid sampling bias. Thus, the included centers were the Medical University of Innsbruck, Tyrol, Austria (Innsbruck), the Medical University of Vienna, Vienna, Austria (Vienna), the Medical University of Graz, Styria, Austria (Graz), the Ospedale dei Bambini “V Buzzi,” Milan, Italy (Milan), and the “IRCCS Burlo Garofolo”, Trieste, Italy (Trieste).

Exclusion criteria of infants of the included centers for the secondary outcome analysis were death, missing MRI or MRI not evaluable due to motion artifacts.

MRI scans of included infants were compared according the allocation in the NIRS group and the control group of the COSGOD III trial. Maternal and neonatal demographic and clinical data were collected during hospital stay as described in Pichler et al. [8].

### Magnetic resonance imaging

Cerebral MRI was performed according to routine protocols of the centers. In Graz and Vienna, MRI was performed before discharge; Innsbruck, Milan and Trieste invited the patients to the outpatient unit at term-equivalent age. One center (Trieste) regularly used sedation because of time constraints for the scan, two centers (Vienna and Milan) only used sedation if strictly necessary and two centers (Innsbruck and Graz) only used a feed-and-wrap protocol without any sedation [9, 13]. The MRI was either performed with a 1.5 Tesla scanner (Graz, Vienna, Trieste, Milano) or a 3 Tesla scanner (Innsbruck). Detailed MRI protocols are available as Additional file 1. During the scan, the SpO_2_ and the HR of the neonate were monitored continuously. The investigation was interrupted at any time if the baby was unstable, or if it was necessary to soothe the baby.

The MRI scans were evaluated by two blinded operators (VN, MH). In cases of discrepancies, consensus was reached through discussion. Brain injuries were categorized as intraventricular hemorrhage, white matter injury, and cerebellar hemorrhage according to Kidokoro et al. [14]. Grade 1 and 2 injuries were classified as mild, while grade 3 and 4 were considered severe. For the diagnosis of brain injury, T1- and T2-weighted images, as well as susceptibility-weighted images were used. The study employed two-dimensional measurements of brain size (i.e., the biparietal diameter, the interhemispheric distance, and the transcerebellar diameter) as parameters of brain growth. These measurements were assessed in T1-weighted images, as previously described [15, 16]. Due to differences in the age at MRI scan, the biparietal diameter and the transcerebellar diameter were corrected for postmenstrual age using a linear correction method: corrected value = measured value + the slope * (40 – postmenstrual age on MR imaging), analogous to Kidokoro et al. [10]. According to Kidokoro et al., a reduction in the biparietal diameter was defined as a z-score < −0.5; for the transcerebellar diameter, two thresholds were applied, with reductions defined as < 50 mm and < 47 mm; an abnormal interhemispheric distance was defined as ≥ 4 mm [10, 14].

### Ethical approval

The trial was approved by the human research ethics committee at each participating site: Graz: institutional ethical board of the Medical University of Graz (reference number: 28–456 ex 15/16); Vienna: institutional review board of the Medical University Vienna (reference number: 1823/2017); Innsbruck: institutional ethical board of the Medical University of Innsbruck (reference number: 1048/2017). Trieste: Friuli Venezia Giulia regional ethical comitee (reference number: Parere CEUR 2020-Sper-061), Milano: Comitato Etico Milano Area 1 (reference number 19140/2018).

### Statistical analyses

Data analysis was performed using SAS 9.4 (SAS Institute Inc., Cary, NC, USA). For comparison of two independent groups Student’s t-tests or Mann-Whitney-U-tests were performed. For comparison of more independent groups Kruskal-Wallis-tests were used. *p*-values were adjusted for multiple comparisons by Bonferroni correction. Comparison of categorical data was obtained by using chi-square or Fisher’s exact test. Significance threshold was set at 0.05.

## Results

**Fig. 1:**
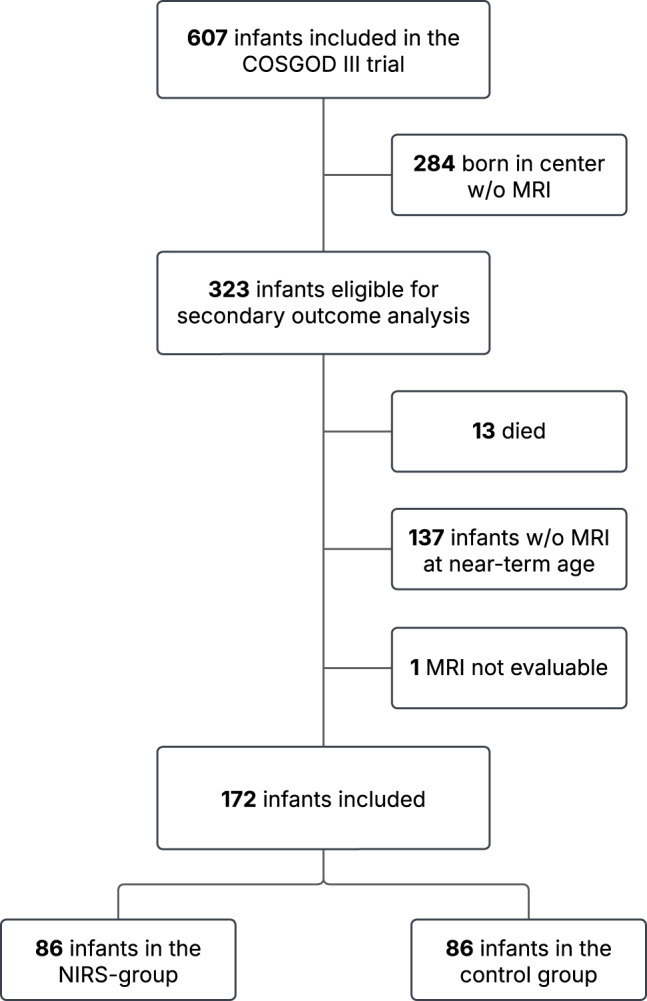
In- and exclusion procedures

Of the 607 infants included in the COSGOD III trial, 323 infants were born in a center participating in this study. Thirteen (4%; NIRS group: *n* = 6, control group *n* = 7) of the 323 neonates died until term-equivalent age. In 172 (55.5%) of the 310 infants, the cerebral MRI was performed and evaluable. 86 (50%) of the included infants were in the NIRS group and 86 (50%) in the control group. Detailed information on the inclusion and exclusion procedure is given in Fig. 1.

The median gestational age of the included infants was 28.4 [IQR 26.4 - 30.4] weeks and the median birth weight was 1045 [IQR 823 - 1363] grams. Maternal and neonatal characteristics of all study participants are displayed in Additional file 2. There was no significant difference in maternal or neonatal characteristics between the NIRS group and the control group, see Table 1.

**Table 1:** Comparison of maternal and neonatal characteristics between the NIRS group and the control group. Values are numbers (percentages) unless stated otherwise

	NIRS group (*n* = 86)	Control group (*n* = 86)	p value
Median (IQR) gestational age (weeks)	28.7 (27.3 - 30.4)	27.6 (26 - 30)	0.069
Median (IQR) birth weight (grams)	1093 (860 - 1370)	974 (810 - 1320)	0.196
Female	35/85 (41.2%)	33/86 (38.4%)	0.708
PPROM	31/85 (36.5%)	32/86 (37.2%)	0.920
Multiple birth	10/84 (11.9%)	6/85 (7.1%)	0.282
Antenatal corticosteroids	85/85 (100%)	83/85 (97.6%)	0.155
Antenatal neuroprotection with magnesium	62/80 (77.5%)	59/78 (75.6%)	0.783
Cesarean section	79/85 (92.9%)	79/86 (91.9%)	0.790
Median (IQR) Apgar score at 5 minutes	9 (8 - 9)	9 (8 - 9)	0.723
Cardiocirculatory resuscitation (first 15 min)	2/83 (2.4%)	3/83 (3.6%)	0.682
Catecholamine use (first 24 hours)	1/83 (1.2%)	0/86 (-)	0.497
Catecholamine use (during initial hospital stay)	9/83 (10.6%)	6/86 (7.0%)	0.404
Surfactant (first 24 hours)	54/86 (62.8%)	63/86 (73.3%)	0.141
Intubation (first 24 hours)	20/81 (24.7%)	21/86 (24.4%)	0.517
Respiratory distress syndrome	81/86 (94.2%)	81/86 (94.2%)	1.000
Early onset sepsis (culture proven)	12/86 (14.0%)	20/86 (23.3%)	0.117
Late onset sepsis (culture proven)	30/86 (34.9%)	42/86 (48.8%)	0.064
Necrotizing enterocolitis	6/86 (7.0%)	2/86 (2.3%)	0.277
Bronchopulmonary dysplasia	12/86 (14.0%)	15/86 (17.4%)	0.529
Retinopathy of prematurity ≥ grade 2	11/86 (12.8%)	18/86 (20.9%)	0.154
Persistent ductus arteriosus with intervention	15/86 (17.4%)	18/86 (20.9%)	0.561

IQR – interquartile range

Time within the predefined crSO₂ target range during the first 15 minutes after birth was 85% (range 0–100%) in the NIRS group. Interventions applied during stabilization and the proportion triggered by a NIRS indication are summarized in Table 2.

**Table 2:** Classification of interventions based on NIRS indication

	NIRS group (*n* = 86)		Control group (*n* = 86)
	Intervention	NIRS indicated	Intervention
Supplemental oxygen	85 (98.8%)	46 (54.1%)	83 (96.5%)
CPAP	83 (96.5%)	13 (15.7%)	84 (97.7%)
PPV	39 (45.3%)	7 (17.9%)	42 (48.8%)
Intubation	5 (5.8%)	2 (40%)	6 (7.0%)
Caffeine	26 (30.2%)	0 (0%)	35 (40.7%)
Catecholamines	1 (1.2%)	1 (100%)	0 (0%)
Surfactant	2 (2.3%)	1 (50%)	4 (4.7%)
Volume	3 (3.5%)	2 (2.3%)	0 (0%)
Others	2 (2.3%)	2 (2.3%)	1 (1.2%)

Median postmenstrual age at MRI was 40.1 [IQR 37.6 - 40.4] weeks. As MRI was performed according to clinical routine, a significant difference for the postmenstrual age at MRI was found between centers (*p* < 0.001). Graz and Vienna usually performed the MRI before discharge, thus the postmenstrual age at MRI was significantly lower than in Innsbruck and Milan. No difference in postmenstrual age at MRI was found between the NIRS group and the control group.

Brain injuries were detected in 61 (36.7%) of all included infants. Most injuries were isolated (*n* = 38, 62.3%). Thirty-one (18.0%) infants were diagnosed with an intraventricular hemorrhage, 28 (17.2%) infants had a white matter injury and in 27 (15.7%) infants a cerebellar hemorrhage was detected. There was no difference in rates of brain injury between the NIRS group and the control group (Table 3).

**Table 3:** Comparison of brain injuries detected by MRI at near-term age between the NIRS group and the control group. Values are numbers (percentages)

	NIRS group (*n* = 86)	Control group (*n* = 86)	p value
Any brain injury	29 (35.4%)	32 (38.1%)	0.715
Any combined brain injury	13 (15.1%)	10 (11.6%)	0.502
White matter disease	17 (21%)	11 (13.4%)	0.200
White matter disease severity			0.354
mild	14 (17.3%)	10 (12.2%)	
severe	3 (3.7%)	1 (1.2%)	
Intraventricular hemorrhage	14 (16.3%)	17 (19.8%)	0.522
Intraventricular hemorrhage severity			0.646
mild	10 (11.6%)	14 (16.3%)	
severe	4 (4.7%)	3 (3.5%)	
Cerebellar hemorrhage	13 (15.1%)	14 (16.3%)	0.834
Cerebellar hemorrhage severity			0.901
mild	8 (9.3%)	10 (11.6%)	
severe	5 (5.8%)	4 (4.7%)	

**Fig. 2:**
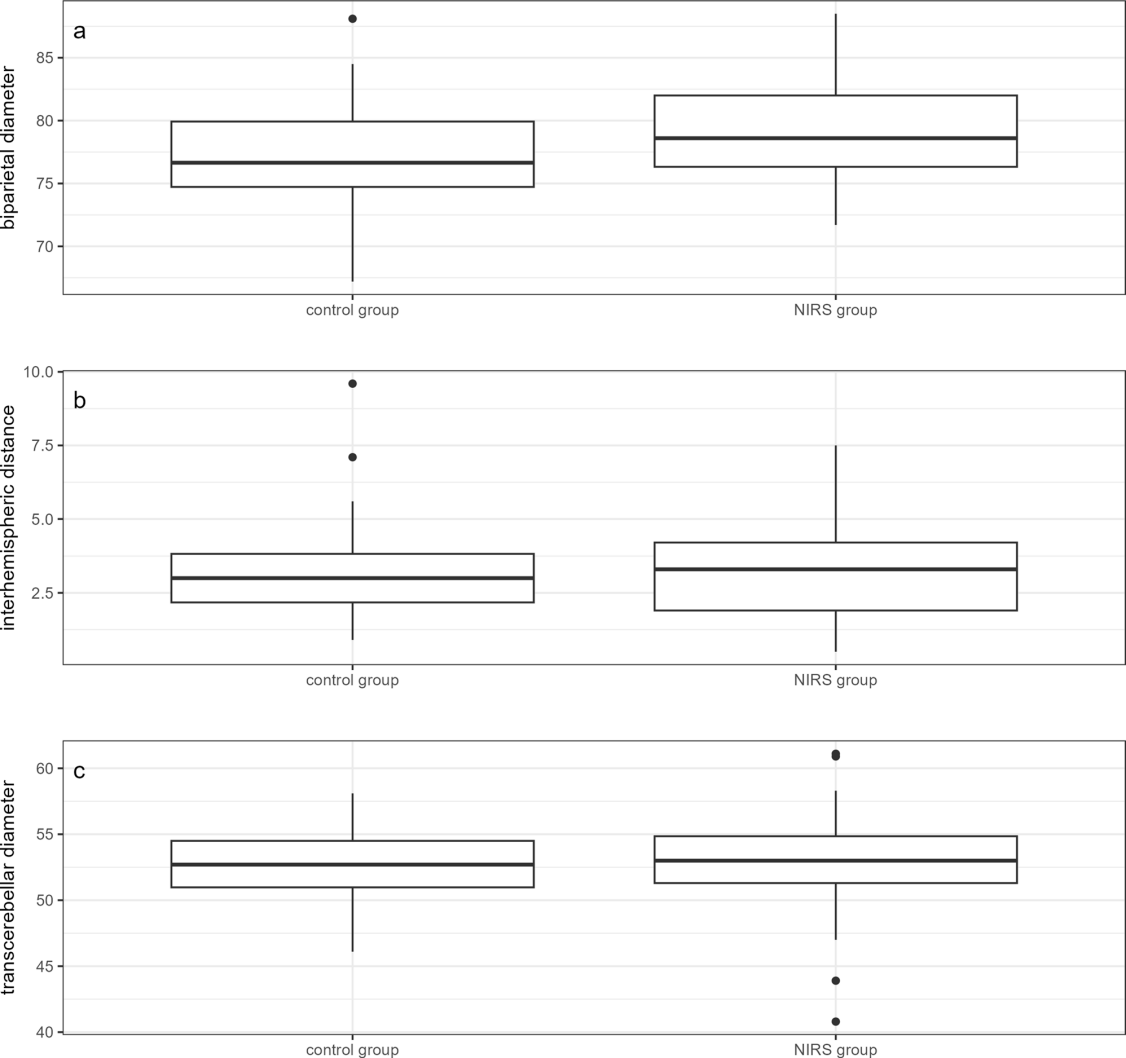
(**a**-**c**) Comparison of brain growth parameters as determined by cerebral MRI at near-term age between the NIRS group and the control group: (**a**) Biparietal diameter, (**b**) Interhemispheric distance, (**c**) Transcerebellar diameter; (median [IQR], centimeters)

Infants of the NIRS group had a larger biparietal diameter than infants in the control group (median [IQR] 78.6 [76.3 - 82.1] vs. 76.7 [74.7 - 80.0] mm, *p* = 0.023). A reduced biparietal diameter (defined as a z-score < −0.5) was observed in 32 (30.2%) infants. A trend toward a higher rate of a reduced biparietal diameter was observed in the control group compared to the NIRS group (*p* = 0.083). Detailed results are shown in Table 4 and Fig. 2a-c.

**Table 4:** Comparison of abnormal brain growth detected by MRI at near-term age between the NIRS group and the control group. Values are numbers (percentages)

	NIRS group (*n* = 86)	Control group (*n* = 86)	p value
Transcerebellar diameter < 50 mm	10/79 (12.7%)	11/80 (13.8%)	0.839
Transcerebellar diameter < 47 mm	2/79 (2.5%)	4/80 (5%)	0.681
Biparietal diameter z-score < −0.5	11/50 (22%)	21/56 (37.5%)	0.083
Interhemispheric distance ≥ 4 mm	16/49 (32.7%)	13/56 (23.2%)	0.281
Biparietal diameter z-score < −0.5 and interhemispheric distance ≥ 4 mm	4/49 (8.2%)	3/56 (5.4%)	0.703

There was no significant difference for the transcerebellar diameter (median [IQR] 52.7 [51.0 - 54.5] vs. 53.0 [51.1 - 54.9] mm, *p* = 0.576) or the interhemispheric distance (median [IQR] 3.0 [2.2 - 3.9] vs. 3.3 [1.9 - 4.2] mm, *p* = 0.634) between the NIRS group and the control group (Fig. 2a-c). An abnormal transcerebellar diameter (defined as < 50 mm) was observed in 21/159 (13.2%) infants, and 29/105 (27.6%) infants had an extended interhemispheric distance (defined as ≥ 4 mm). A combination of a reduced biparietal diameter and an extended interhemispheric distance was found in 7/105 (6.7%) infants. No differences were found between the NIRS group and the control group. Detailed results are shown in Table 4 and Fig. 2-ca

## Discussion

In this secondary outcome analysis of the COSGOD III trial, we investigated the effect of crSO_2_-guided interventions during the immediate postnatal transition of very preterm infants on the incidence of brain injury and on brain growth as assessed by near-term MRI. Our analyses demonstrated that the intervention did not result in statistically significant differences in the incidence of brain injury between the NIRS group and control group.

Observational studies suggest that cerebral hypoxia is a risk factor for intraventricular hemorrhage in preterm infants [17, 18]. While these studies underscore the critical importance of managing cerebral oxygenation during the immediate postnatal period, two recent randomized controlled trials on crSO_2_-guided interventions in very and extremely preterm infants did not result in substantially higher survival without (severe) cerebral injury as detected by ultrasound [8, 19]. The lower sensitivity of ultrasound in detecting certain types of preterm brain injury may partly explain the lack of a significant effect. MRI has been demonstrated to be more effective than ultrasound in detecting a broader spectrum of preterm brain injury including cerebellar hemorrhage and non-cystic white matter injury [9]. The rates of cerebral injury detected by MRI in our cohort were within the ranges expected in comparable populations [14, 20]. In the current study, crSO_2_-guided interventions during immediate postnatal transition did not lead to a reduction in the incidence of either injury type. Similarly, the use of crSO_2_-guided interventions for the first 72 h after birth did also not show a benefit with regard to cerebral injury in preterm infants < 28 gestational weeks assessed by MRI at term-equivalent age [21].

It is conceivable that the most vulnerable period for brain injury extends beyond the initial resuscitation phase, and prolonged intervention may be necessary to achieve neuroprotective effects. Especially circulatory factors and severe respiratory problems have been proposed to play a role in the onset of cerebellar hemorrhage [9, 22, 23]. Similarly, infection, inflammation as well as hypoxia-ischemia have been identified as key contributors to the multifactorial pathogenesis of white matter injury [24, 25]. Even though the critical importance of the immediate fetal-to-neonatal transition in preterm infants is beyond question in the onset of brain injury, the impact of the intervention during the initial 15-minute period may be outweighed by complications that arise during the subsequent weeks spent in the neonatal intensive care unit. Furthermore, it may be speculated that the study’s organization, which necessitated antenatal parental consent and the availability of the research team, resulted in the selection of infants born under ‘optimal conditions’, potentially excluding those born under more urgent circumstances (e.g., chorioamnionitis). Therefore, it is conceivable that infants who may benefit most from crSO_2_-guided oxygen administration were underrepresented in the study cohort.

Despite the fact that the trial intervention did not result in a reduction in the incidence of brain injury, infants in the NIRS group exhibited a significantly larger biparietal diameter compared with controls group. No statistically significant differences were observed in the transcerebellar diameter or the interhemispheric distance. The biparietal diameter has been proposed as an easily measurable surrogate for brain volume and as such has been associated with long-term outcomes [10, 14, 26]. While the current evidence does not emphasize the predictive significance of the biparietal diameter as an individual measure in relation to neurodevelopmental outcomes, the predictive value increases when using population-based z-scores [16]. In this respect, a trend toward a lower incidence of infants exhibiting a biparietal diameter z-score < −0.5 was observed in the NIRS group, suggesting a potential benefit in brain growth. While these initial results may be considered favorable, it is important to exercise caution and refrain from overinterpretation. The difference was modest, its clinical significance remains uncertain, and measurements were not available for the entire cohort due to differences in imaging protocols across centers. Nevertheless, the relationship between oxygen delivery and brain growth remains a critical area of research. Preterm infants are at high risk of impaired brain growth and adverse neurodevelopmental outcomes, and cerebral hypoxia has been identified as a key risk factor [27, 28]. Further studies using volumetric and microstructural MRI analyses are required to validate our findings. The employment of advanced imaging techniques, incorporating volumetric analysis or artificial intelligence-based image evaluation, holds promise for detecting alterations in unexpected areas beyond the predefined regions of interest. These techniques have the potential to provide a more detailed characterization of the impact of cerebral crSO_2_-guided interventions during the fetal-to-neonatal transition on brain development.

This study has several strengths, including its multicenter design and the uniform review of the MRI scans by two independent blinded experts. The latter addressed the presence of certain methodological limitations, including variations in MRI protocols across the different research centers.

In addition, MRI at term-equivalent age was performed only in a subset of participating centers, which limits our analysis to infants with available imaging data. This selective inclusion may introduce bias and should be considered when interpreting the observed differences in brain growth, as the findings may not be fully generalizable to the entire COSGOD III population.

In conclusion, CrSO_2_-guided interventions during the fetal-to-neonatal transition in very preterm infants did not show a benefit regarding brain injury rates as determined by MRI at near-term age. While differences in structural brain growth were observed, these were modest, and to the subset of infants with a complete MRI dataset. Prospective neurodevelopmental follow-up is needed to determine whether these early structural differences translate into meaningful functional benefits.

## Electronic supplementary material

Below is the link to the electronic supplementary material.

Supplementary Material 1

Supplementary Material 1

## Data Availability

Data are available on reasonable request.
