# Supplementary material for: Impact of cerebral oxygenation-guided resuscitation during immediate postnatal transition on brain injury and brain growth detected by MRI in very preterm neonates: a secondary outcome analysis of the multicenter randomized phase 3 clinical COSGOD III trial
Source: Ital J Pediatr. 2026 Feb 24;52:49. doi: 10.1186/s13052-026-02216-7 (PMC13037069; doi:10.1186/s13052-026-02216-7)
Supplement: Supplementary file 1 — Supplementary Material 1 [file 13052_2026_2216_MOESM1_ESM.docx]

**Detailed MRI protocols for each center**

|  | **Innsbruck** | **Graz** | **Vienna** | **Trieste** | **Milano** |
| --- | --- | --- | --- | --- | --- |
| **Sedation** | feed-and-wrap,  no sedation | feed-and-wrap,  no sedation | sedation  if strictly necessary | sedation  regularly | sedation if  strictly necessary |
| **Scanner** | Siemens Magnetom Skyra | Siemens Magnetom Sola | Philips Ingenia MR system | Philips Ingenia MR system |  |
| **Field strength** | 3.0 Tesla | 1.5 Tesla | 1.5 Tesla | 1.5 Tesla | 1.5 Tesla |
| **T1** | 3D MP-RAGE  covering the whole head matrix 144 × 192, TE 4.54 ms, TR 1770 ms, TI 1,000 ms, flip angle 9 degrees, FOV 20 × 15 cm,  slice thickness: 1.0 mm, gap: 0.5 mm | 3D MPR axial  covering the whole head matrix 192 x 192, TR 2340 ms, TE 2.7 ms,  FOV 20 cm x 20 cm,  slice thickness: 1.0 mm | T1 SE axial  matrix 144 × 115, TE 15 ms, TR 400 ms, voxel size 0.83 × 1.05 × 3.00 mm, FOV 120 × 120 × 90 mm;  T1 3D sagittal  matrix 160 × 160, TE 7.6 ms, TR 25 ms, voxel size 0.75 × 0.75 × 2.00 mm, FOV 120 × 120 × 99 mm | T1 SE sagittal  matrix 176 × 177, TE 15 ms, TR 400 ms, voxel size 0.85 × 0.85 × 4.00 mm, FOV 150 × 150 × 84 mm;  T1 IR axial  matrix 212 × 110, TE 15 ms, TR/TI 2084/400 ms, voxel size 0.70 × 0.99 × 3.00 mm, FOV 120 × 120 × 99 mm  T1 IR coronal  matrix 212 × 110, TE 15 ms, TR/TI 2331/400 ms, voxel size 0.70 × 0.99 × 3.00 mm, FOV 149 × 109 × 102 mm; | T1 SE axial  matrix 156 × 102, TE 12 ms, TR 750 ms, voxel size 1.04 × 1.13 × 3.00 mm, FOV 150 mm  T1 SE sagittal  matrix 190 × 190, TE 12 ms, TR 450 ms, voxel size 0.8 × 0.8 × 3.00 mm, FOV 150 mm |
| **T2** | axial  TE: 99 ms, TR: 4590 ms, FOV: 15 x 11 cm, matrix: 147 x 256, slice thickness: 3 mm, no gap | axial  FOV 15 x 15 cm, matrix 256 x 256, TR 4830 ms, TE 98 ms, 3 mm slice thickness | axial, sagittal, and coronal turbo spin-echo (TSE), matrix 128 × 113 to 116 × 103, TE 140 ms, TR 3,000 ms, voxel size 0.94 × 1.06 × 3.00 mm, FOV 110–120 × 110–120 × 102–108 mm | axial and coronal  turbo spin-echo (TSE-HR), axial : matrix 228 × 160, TE 200 ms, TR 5720 ms, voxel size 0.65 × 0.69 × 3.00 mm, FOV 149 × 110 × 89 mm; coronal: matrix 228 × 160, TE 200 ms, TR 6443 ms, voxel size 0.65 × 0.69 × 3.00 mm, FOV 149 × 110 × 102 mm | axial and coronal  turbo spin-echo (TSE), matrix 244 × 126, TE 200 ms, TR 6000 ms, voxel size 0.7 × 0.8 × 3.00 mm, FOV 170 mm |
| **SWI** | matrix 182 × 256, TE 20 ms, TR 27 ms, FOV 20 × 15 cm, slice thickness: 2.0 mm, gap: 0.4 mm | FOV 20 cm x 20 cm, matrix 256 x 256, TR 50 ms, TE 40 ms)  slice thickness: 2.0 mm | matrix 200 × 138, TE 12 ms, TR 51 ms, voxel size 0.85 × 1.00 × 2.00 mm, FOV 170 × 139 × 90 mm | matrix 152 × 123, TR 23 ms, TR 33 ms, voxel size 0.99 × 1.00 × 2.00 mm, FOV 150 × 123 × 90 mm | matrix 212 × 150, TE1 11 ms, TR 51 ms, delta TE 11.0 ms, voxel size 0.8 × 0.8 × 3.00 mm, FOV 170 |
