# Supplementary material for: Impact of cerebral oxygenation-guided resuscitation during immediate postnatal transition on brain injury and brain growth detected by MRI in very preterm neonates: a secondary outcome analysis of the multicenter randomized phase 3 clinical COSGOD III trial
Source: Ital J Pediatr. 2026 Feb 24;52:49. doi: 10.1186/s13052-026-02216-7 (PMC13037069; doi:10.1186/s13052-026-02216-7)
Supplement: Supplementary file 2 — Supplementary Material 1 [file 13052_2026_2216_MOESM2_ESM.docx]

***Additional Table.*** *Maternal and neonatal characteristics of study participants. Values are numbers (percentages) unless stated otherwise.*

|  | All (n = 172) | Innsbruck (n = 65) | Vienna (n = 46) | Milan (n = 31) | Graz (n = 24) | Triest (n = 6) | p value |
| --- | --- | --- | --- | --- | --- | --- | --- |
| Median (IQR) gestational age (weeks) | 28.4 (26.4 - 30.4) | 29.9 (28.1 - 31.1)**^V,G^** | 26.6 (25.9 - 27.4)**^I,M,Tr^** | 30.0 (28.6 - 30.9)**^V,G^** | 26.5 (24.9 - 28.9)**^I,M,T^** | 30.4 (29.7 - 31.1)**^V,G^** | **<0.001** |
| Median (IQR) birth weight (grams) | 1045 (823 - 1363) | 1300 (1000 - 1495)**^V,G^** | 860 (770 - 990)**^I,M,Tr^** | 1077 (900 - 1450)**^V^** | 850 (668 - 1164)**^I^** | 1215 (1160 - 1400)**^V^** | **<0.001** |
| Female | 68 (39.8%) | 22 (33.8%) | 19 (41.3%) | 15 (50%) | 10 (41.7%) | 2 (33.3%) | 0.652 |
| PPROM | 63 (36.8%) | 21 (32.3%) | 25 (54.3%) | 8 (26.7%) | 8 (33.3%) | 1 (16.7%) | 0.065 |
| Multiple birth | 16 (9.5%) | 4 (6.2%) | 2 (4.4%) | 8 (27.6%) | 2 (8.3%) | 0 (-) | **0.022** |
| Antenatal corticosteroids | 168 (98.8%) | 63 (98.4%) | 46 (100%) | 30 (100%) | 23 (95.8%) | 6 (100%) | **<0.001** |
| Antenatal neuroprotection with magnesium | 121 (76.6%) | 44 (74.6%) | 44 (97.8%) | 14 (46.7%) | 15 (83.3%) | 4 (66.7%) | **0.006** |
| Cesarean section | 158 (92.4%) | 65 (100%) | 41 (89.1%) | 25 (83.3%) | 21 (87.5%) | 6 (100%) | 0.314 |
| Median (IQR) Apgar score at 5 min | 9 (8 - 9) | 8 (8 - 9)**^V^** | 9 (9 - 9)**^I^** | 9 (8 - 9) | 8 (8 - 9) | 9 (8 - 9) | **0.007** |
| Cardiocirculatory resuscitation (first 15 min) | 5 (3%) | 2 (3.1%) | 1 (2.2%) | 1 (3.6%) | 0 (-) | 1 (16.7%) | 0.314 |
| Catecholamine use (first 24 hours) | 15 (8.8%) | 1 (1.5%) | 12 (26.1%) | 0 (-) | 2 (8.3%) | 0 (-) | **<0.001** |
| Surfactant (first 24 hours) | 117 (68%) | 55 (84.6%) | 26 (56.5%) | 13 (41.9%) | 20 (83.3%) | 3 (50%) | **<0.001** |
| Intubation (first 24 hours) | 41 (24.0%) | 15 (23.1%) | 16 (34.8%) | 3 (10.3%) | 4 (16.7%) | 3 (75%) | 0.052 |
| Respiratory distress syndrome | 162 (94.2%) | 61 (93.8%) | 45 (97.8%) | 30 (96.8%) | 23 (95.8%) | 3 (50%) | **0.009** |
| Sepsis (culture proven) | 72 (41.9%) | 22 (33.8%) | 33 (71.7%) | 12 (38.7%) | 5 (20.8%) | 0 (-) | **<0.001** |
| Necrotizing enterocolitis | 8 (4.7%) | 3 (4.6%) | 5 (10.9%) | 0 (0%) | 0 (0%) | 0 (-) | 0.193 |
| Bronchopulmonary dysplasia | 27 (15.7%) | 4 (6.2%) | 14 (30.4%) | 2 (6.5%) | 7 (29.2%) | 0 (-) | **0.001** |
| Retinopathy of prematurity ≥ grade 2 | 29 (16.9%) | 3 (4.6%) | 18 (39.1%) | 0 (-) | 8 (33.3%) | 0 (-) | **<0.001** |
| Persistent ductus arteriosus with intervention | 33 (19.2%) | 4 (6.2%) | 21 (45.7%) | 5 (16.1%) | 3 (12.5%) | 0 (-) | **<0.001** |

*Superscript letters indicate significant differences after post-hoc analysis. G – Graz; I – Innsbruck; M – Milan; T – Triest; V – Vienna; IQR – interquartile range;*
